# A Cross-Sectional Study Assessing the Contributions of Body Fat Mass and Fat-Free Mass to Body Mass Index Scores in Male Youth Rugby Players

**DOI:** 10.1186/s40798-018-0130-7

**Published:** 2018-05-02

**Authors:** Olivier Gavarry, Gregory Lentin, Patrick Pezery, Anne Delextrat, Guillaume Chaumet, Alain Boussuges, Julien Piscione

**Affiliations:** 1 UFR STAPS, Université de Toulon, BP 20132, 83957 La Garde Cedex, France; 2 UMR MD2 Dysoxie-Suractivité, IFR Jean Roche, Faculté de Médecine, Université Aix-Marseille, France-Institut de Recherche Biomédicale des Armées (IRBA), Brétigny-sur-Orge, France; 3 Sport and Health Science Department, Oxford Brookes University, Oxford, UK; 4 Altrabio, SA, Lyon, France; 5 Département Recherche et Développement, Fédération Française de Rugby, 3-5 rue Jean de Montaigu, 91463 Marcoussis, France

**Keywords:** Health, Children, Obesity, Rugby union, Body fat mass index

## Abstract

**Background:**

In some sports such as rugby, a large body size is an advantage, and the desire to gain weight can bring young players to become overweight or obese. The aim of this study was to evaluate the prevalence of overweight and obesity and the contribution of body fat mass index (BFMI) and fat-free mass index (FFMI) to body mass index (BMI) changes among young male rugby players (15-a-side rugby).

**Methods:**

The criteria of the International Obesity Task Force were used to define overweight and obesity from BMI. The method of skinfold thickness was used to assess percentage of body fat (%BF), BFMI, and FFMI. Excess body fat was defined by using BFMI and %BF above the 75th percentile. Data were grouped according to the age categories of the French Rugby Federation (U11, under 11 years; U13, under 13 years; U15, under 15 years) and to BMI status (NW normal-weight versus OW/OB overweight/obese).

**Results:**

Overall, 32.8% of the young players were overweight, and 13.8% were obese. However, 53% of young players classified as obese and overweight by BMI had an excess body fat by using BFMI above the 75th percentile. FFMI increased significantly between U11 and U13 in both groups, without significant change in BMI and BFMI. Both groups had similar significant gains in BMI and FFMI between U13 and U15, while BFMI only increased significantly in OW/OB (+ 18.5%). The strong correlations between BMI and %BF were systematically lower than those between BMI and BFMI. FFMI was strongly or moderately associated with BFMI.

**Conclusions:**

Chart analysis of BFMI and FFMI could be used to distinguish changes in body composition across age categories in young male rugby players classified as normal-weight, overweight, and obese by BMI.

## Key Points

- The prevalence of overweight included obesity (IOTF criteria) was high (46.6%) in young male rugby players aged 9–14 years. However, 53% of young players classified as obese and overweight by BMI had an excess body fat by using BFMI above the 75th percentile.
- Calculating BMI for young male rugby players in clubs to detect overweight and obesity must be used with caution due to significant moderate and strong correlations between BMI, BFMI, and FFMI and to different combinations of FFMI and BFMI that result in the same BMI.
- There is a need to include the evaluation of body composition using BFMI and FFMI chart analysis to avoid misclassification of some young male rugby players and to design individual intervention for fat loss or muscle gain.

## Background

Increasing spontaneous physical activity and sports participation in pediatric populations is an important recommendation in the prevention and the treatment of obesity [1]. However, the benefits of sports participation have not been systematically demonstrated in the prevention of obesity in the youth [2]. In addition, although a large number of studies have evaluated the prevalence of childhood and adolescent obesity in sedentary children, there is a paucity of research in young athletes [3–6]. In some sports such as rugby, a large body size is an advantage, and the desire to gain weight can bring young players to become overweight or obese. In another contact sport, Malina et al. [4] observed a high prevalence of overweight and obesity among young American football players (42.6%). This increase in adolescence obesity could translate later into an even greater prevalence of adulthood obesity [7]. Moreover, overweight and obesity in adolescents may contribute to a greater risk of injury while playing sports [8] due to persistent orthopedic problems and musculoskeletal pain in the lower limb [9]. In addition, childhood and adolescence obesity, characterized by reduced muscular strength in the lower limb [10] and lower aerobic fitness [11], can both result in a decrease in athletic performances.

In rugby union as in many other sports, competitions are organized according to age categories in children and adolescents to take into account the influence of growth on anthropometric characteristics and consequently performance. Several studies, conducted in the UK and Australia, focused on anthropometric characteristics in adolescent rugby players, taking into consideration the playing level and position [12, 13], physical performances [13, 14], ethnicity [12], and role in a team [15]. However, no data are available about changes in body composition in a large sample of young rugby players during pubescence. In addition, the majority of these studies in this area used body mass index (BMI) and/or percentage of body fat (%BF), which are not considered as the most relevant indexes of an excess of fat mass during growth [16]. Finally, the few studies investigating the prevalence of overweight and obesity in young athletes did not assess body composition [3–6]. Indeed, BMI is easily measurable and commonly used to classify children or adolescents as obese or overweight. However, BMI does not distinguish between fat and lean body mass, while obesity is defined as an excess body fat [17]. In the same way, using %BF is questionable due to its inability to take into account height, lean body mass, or body proportions that markedly change during growth [16]. For the reasons, VanItalie et al. [18] have recommended to use the two components of BMI as indicators of nutritional status: the body fat mass index (BFMI) and the fat-free mass index (FFMI). Thus, the contributions of body fat mass and fat-free mass to BMI scores and excess adiposity can be determined by chart analysis [19].

In this context, the purposes of this study were (1) to assess the prevalence of overweight and obesity by using BMI in young male rugby players in order to identify the proportion of obese and overweight players having an excess adiposity by using BFMI above the 75th percentile and (2) to examine the contribution of body fat mass index (BFMI) and fat-free mass index (FFMI) to the changes in BMI across different age categories. This cross-sectional study also aimed to evaluate the differences in body composition between normal-weight and overweight/obese players. The relations between BMI, BFMI, FFMI, and %BF have been studied. We used the age categories of the French Rugby Federation in our analysis in order to reflect on differences in body composition within the constraints of real competition.

## Methods

### Participants and Data Collection

A total of 1000 young male rugby players involved in playing 15-a-side rugby from a population of 4442 (age 9–14 years) located in the Provence-Côte d’Azur rugby county (France) were included in a cross-sectional study conducted between September 2012 and May 2013. From these children, 738 volunteered to participate in the study and all the players were attached to clubs. Informed consent was obtained from child and parent or legal guardian prior to each child’s participation. The study was approved by the Ethics Committee of the University of Toulon and was conducted in accordance with the Declaration of Helsinki. The young rugby players were divided into three groups according to age categories set by the French Rugby Federation: under 11 years (U11; 10.0 ± 0.6 years, *n* = 247), under 13 years (U13; 12.1 ± 0.5 years, *n* = 261), and under 15 years (U15; 14.0 ± 0.5 years, *n* = 230). Age was recorded as a decimal value for each child using their date of birth and the date of testing. Height and body mass were measured to the nearest 0.1 cm and 0.1 kg, respectively, using a portable stadiometer (Leicester high measure, Tanita, UK) and an electronic weighing scale (SECA 920, class 3, Germany) with participants wearing light exercise clothes without shoes. Consequently, BMI was calculated as body mass (kg) divided by height squared (m^2^).

### Evaluation of Body Composition

Body composition was assessed by the skinfold thickness method at selected sites with a Harpenden skinfold caliper (Baty International, England). All skinfolds were measured by a single experienced researcher to eliminate inter-tester variability. The test-retest intraclass coefficients on a random sample of 50 subjects in each age category were greater than 0.99. Three measurements of each site were taken. If these three values varied by more than 0.2 mm, an additional measurement was taken. The mean of skinfold measurements at each site was used for statistical analysis. The protocol for precise skinfold location and measurement was carefully followed, according to the standardized procedures and guidelines described by Lohman [20]. According to the equations of Durnin and Rahaman [21], percentage body fat and fat-free mass were determined: body density (BD) = 1.1533–0.0643 × log sum of four skinfolds (triceps, biceps, subscapular, suprailiac), %BF = (4.95/BD − 4.5) × 100. Fat mass and fat-free mass were then expressed in kilograms in order to calculate BFMI (kg m^−2^) and FFMI (kg m^−2^) making it possible to adjust body composition to height [20], BMI (kg m^−2^) = BFMI (kg m^−2^) + FFMI (kg m^−2^).

### Classification of Young Male Rugby Players

The prevalence of overweight and obesity was determined using the IOTF (International Obesity Task Force) criteria [17]. These criteria have been used in a previous study [4] to classify young players as normal-weight players (NW) and overweight/obese players (OW/OB), across age categories.

Excess body fat in young male rugby players was defined by using BFMI and %BF above the 75th percentile as previously described by Weber et al. [22], with the use of our reference data for BFMI (U11, > 5.6; U13, > 5.7; U15, > 8.2 kg m^−2^) and for %BF (U11, > 26.1; U13, > 27.3; U15, > 30%).

### Statistical Analysis

The statistical analysis was performed using Statistica 6.1. (Statsoft, Inc. 1984–2003). The prevalence of obesity and overweight and 95% confidence interval (CI) were calculated. A chi-square test was used to evaluate the distributions of overweight and obesity by age category. Parameters studied had a normal distribution as assessed by the Kolmogorov-Smirnov test. A two-way analysis of variance (ANOVA) was used to compare BMI, BFMI, and FFMI values between different age categories (U11, U13, U15) and between groups (normal-weight versus overweight/obese players). Post hoc comparisons were made using Scheffe’s test. Effect sizes were calculated using partial eta squared (η2p) and Cohen’s d. Pearson correlation coefficients were calculated to examine the relationship between BMI, BFMI, and FFMI. Statistical significance was set at *p* < 0.05.

## Results

The prevalence of overweight and obesity and 95%CI by age category are presented in Table 1. Similar prevalence of overweight and obesity was observed in U11 and U13. However, the prevalence of overweight and obesity increased drastically between U13 and U15 (overweight, + 14 pts.; obesity, + 19.1 pts.; overweight and obesity, + 33.1 pts.).

**Table 1:** Prevalence of overweight and obesity in young rugby players by age category (IOTF criteria)

	Overweight		Obese		Overweight and obese	
	%	95% CI	%	95% CI	%	95% CI
IOTF						
All	32.8	29.4–36.2	13.8	11.3–16.3	46.6	43–50.2
Under 11 years	23.9	18.6–29.2	9.7	6.0–13.4	33.6	27.7–39.5
Under 13 years	30.3	24.7–35.8	7.3	4.1–10.4	37.5	31.7–43.4
Under 15 years	45.2	38.8–51.6	25.7	20–31.3	70.9	65–76.7

*95% CI* 95% confidence interval limits

Normal-weight, overweight, and obese rugby players (IOTF criteria) were classified as having excess body fat by BFMI and %BF above the 75th percentile (Table 2). For BFMI > 75th percentile, a very low percentage of normal-weight players in U11 (0.6%) and U13 (1.8%) was in this category and none in U15. 65.5% (U11), 57% (U13), and 6.7% (U15) of overweight players and 100% (U11), 94.4% (U13), and 89.4% (U15) of obese players had an excess body fat. For %BF > 75th percentile, 3.6% (U11), 4.9% (U13), and 0% (U15) of normal-weight players; 58.6% (U11), 50.6% (U13), and 15.4% (U15) of overweight players; and 95.6% (U11), 94.4% (U13), and 71.2% (U15) of obese players had an excess body fat.

**Table 2:** Percentage of normal-weight, overweight, and obese young male rugby players (IOTF criteria) having an excess body fat classified by BFMI and %BF above the 75th percentile

	Under 11 years			Under 13 years			Under 15 years		
	NW	OW	OB	NW	OW	OB	NW	OW	OB
BFMI > 75th percentile	0.6	65.5	100	1.8	57.0	94.4	0	6.7	89.4
%BF > 75th percentile	3.6	58.6	95.6	4.9	50.6	94.4	0	15.4	71.2

*NW* normal-weight, *OW* overweight, *OB* obese, *BFMI* body fat mass index, *%BF* percentage of body fat

**Fig. 1:**
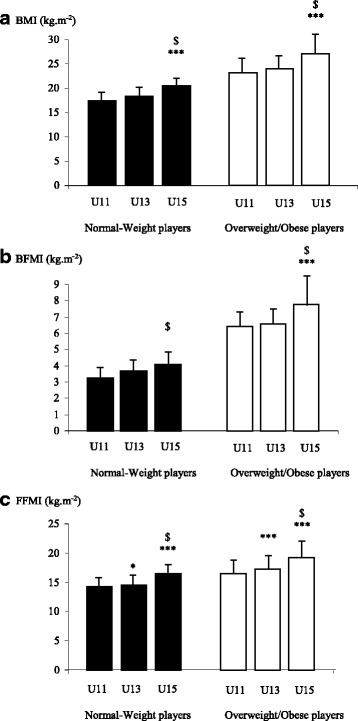
Changes in body mass index (BMI), body fat mass index (BFMI), and fat-free mass index (FFMI) between age categories (U11, under 11 years; U13, under 13 years; U15, under 15 years) in normal-weight (NW) and overweight and obese (OW/OB) young male rugby players. Notes: data presented as mean ± SD. *p < 0.05 and ***p < 0.001 significant differences from previous age category. ^$^*p* < 0.001 significant differences between U11 and U15. BMI (**a**): main effect for category—*p* < 0.001, η^2^_p_ 0.261; main effect for group—*p* < 0.001, η^2^_p_ 0.578; interaction category × group—not significant. BFMI (**b**): main effect for category—*p* < 0.001, η^2^_p_ 0.081; main effect for group—*p* < 0.001, η^2^_p_ 0.531; interaction category × group—*p* = 0.02, η^2^_p_ 0.010. FFMI (**c**): main effect for category—*p* < 0.001, η^2^_p_ 0.363; main effect for group—*p* < 0.001, η^2^_p_ 0.475; interaction category × group—not significant

For both groups, no significant difference was found in BMI between U11 and U13 whereas BMI increased significantly between U13 and U15 (NW: + 2.4 kg m^−2^/+ 13.2%, 95% CI 3.2 to 1.6, *d* = 1.26, *p* < 0.001; OW/OB: + 3.2 kg m^−2^/+ 13%, 95% CI 4.5 to 1.8, *d* = 0.88, *p* < 0.001; Fig. 1a). The significant interaction for BFMI is presented in Fig. 1b. In normal-weight players, BFMI did not change significantly across age categories. In overweight/obese players, BFMI only increased significantly between U13 and U15 (+ 1.2 kg m^−2^/+ 18.5%, 95% CI 2.0 to 0.4, *d* = 0.6, *p* < 0.001). For both overweight/obese and normal-weight players, FFMI increased significantly between U11 and U13 (NW: + 0.4 kg m^−2^/+ 2.8%, 95% CI 0.8 to 0.0, *d* = 0.40, *p* < 0.001; OW/OB: + 0.7 kg m^−2^/+ 6%, 95% CI 1.5 to 0.2, *d* = 0.46, *p* = 0.02) and between U13 and U15 (NW: + 2 kg m^−2^/+ 13.5%, 95% CI 2.4 to 1.5, *d* = 0.54, *p* < 0.001; OW/OB: + 2 kg m^−2^/+ 18.3%, 95% CI 2.7 to 1.2, *d* = 1.05, *p* < 0.001; Fig. 1c).

Obese/overweight players had a significantly (*p* < 0.001) greater BMI (U11: + 5.6 kg m^−2^, 95% CI 6.1 to 5.1, *d* = 1.69; U13: + 5.7 kg m^−2^, 95% CI 6.1 to 5.1, *d* = 1.67; U15: + 6.4 kg m^−2^, 95% CI 7.2 to 5.5, *d* = 1.53), BFMI (U11: + 3.2 kg m^−2^, 95% CI 3.5 to 2.8, *d* = 1.70; U13: + 3.0 kg m^−2^, 95% CI 3.2 to 2.6, *d* = 1.65; U15: + 3.7 kg m^−2^, 95% CI 4.2 to 3.2, *d* = 1.52), and FFMI (U11: + 2.4 kg m^−2^, 95% CI 2.7 to 2.2, *d* = 1.54; U13: + 2.7 kg m^−2^, 95% CI 3.0 to 2.4, *d* = 1.34; U15: + 2.7 kg m^−2^, 95% CI 3.2 to 2.3, *d* = 1.35) than normal-weight players in each age category. The effect size for difference was large.

As shown in Table 3, in normal-weight players, BMI was moderately associated with BFMI (*r* = 0.71–0.75, *p* < 0.001) and with FFMI (*r* = 0.66–0.75, *p* < 0.001) in U11 and U15 but strongly associated with BFMI (*r* = 0.85, *p* < 0.001) and with FFMI (*r* = 0.86, *p* < 0.001) in U13. In overweight/obese players, BMI was strongly associated with BFMI (*r* = 0.81–0.92, *p* < 0.001) in all age categories. In contrast, the association between BMI and FFMI was weak (*r* = 0.33, *p* < 0.01), moderate (*r* = 0.72, *p* < 0.001), and strong (*r* = 0.90, *p* < 0.001) in U11, U13, and U15, respectively. The correlations between BMI and %BF were systematically lower than those observed between BMI and BFMI (Table 3).

**Table 3:** Pearson correlation coefficients between body mass index, body fat mass index, fat-free mass index, and percentage of body fat in young male rugby players

	BFMI (kg m^−2^)	FFMI (kg m^−2^)	%BF
Under 11 years			
Overall	0.96***	0.94***	0.87***
Normal-weight	0.75***	0.66***	0.64***
Overweight/obese	0.89***	0.34**	0.74***
Under 13 years			
Overall	0.91***	0.90***	0.79***
Normal-weight	0.85***	0.86***	0.66***
Overweight/obese	0.81***	0.72***	0.35**
Under 15 years			
Overall	0.95***	0.93***	0.82***
Normal-weight	0.71***	0.75***	0.54***
Overweight/obese	0.92***	0.90***	0.69***

*BFMI* body fat mass index, *FFMI* fat-free mass index, *%BF* percentage of body fat

**p < 0.01; ***p < 0.001

**Fig. 2:**
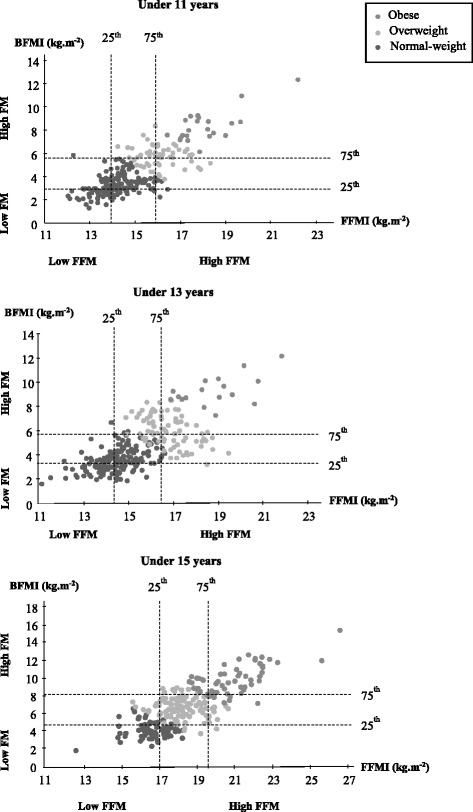
Levels of fat-free mass index (FFMI) versus body fat mass index (BFMI) according to the 25–75th percentile distribution among young male rugby players by age category. Notes: FM, fat mass; FFM, fat-free mass. Low FM < 25th BFMI percentile; high FM > 75th BFMI percentile; low FFM < 25th FFMI percentile; high FFM > 75th FFMI percentile

Figure 2 shows levels of FFMI and BFMI for each age category in young rugby players. FFMI was strongly associated with BFMI in U11 (*r* = 0.80, *p* < 0.001) and moderately associated with BFMI in U13 (*r* = 0.66, *p* < 0.001) and in U15 (*r* = 0.77, *p* < 0.001).

## Discussion

To date, no information is available in the literature on body composition assessed from height-normalized indexes of the body’s fat-free mass and fat mass in a large sample of young rugby players. The use of the two components of BMI (BFMI and FFMI) makes it possible to assess whether BMI differences between age categories are linked to the variations of fat mass or fat-free mass [18] and to calculate the proportion of players classified as normal-weight, overweight, and obese by BMI having an excess adiposity by BFMI above the 75th percentile [22].

Overall, the prevalence of overweight and obesity among 9- to 14-year-old male rugby players was high (46.6% IOTF criteria) and exceeded the values of national studies in France and in Europe. In the present study, 32.8% of the young rugby players were overweight and 13.8% were obese using the IOTF classification. Our results differ from that observed in the general population at this age in France. In a representative sample of French children aged 10–15 years [23, 24], the prevalence of overweight including obesity was considerably lower (17.5 to 19.1% in 2004–2005) to that reported in our study. To our knowledge, the comparison of our results with those from other sports is difficult because of the paucity of studies in this area [3–6]. It is interesting that the study of Malina et al. [4] has reported a similar prevalence (42.6%) to our study in young American football players.

Identifying key periods during growth for the development of excess of body fat in young rugby players may allow us to develop strategies for the prevention and treatment of this condition. According to our data, it is important to distinguish between normal weight and overweight/obese players to track body fatness across age categories in this population, as indicated by the significant interaction observed (Fig. 1b). Indeed, for normal-weight players, BFMI did not change significantly during the transition between age categories. For overweight/obese players, the significant increase in BFMI was only observed during the transition between U13 and U15 (+ 18.5%). The same result was noted for the variation in BMI in this group. However, our data clearly demonstrated a significant increase in body fat in young rugby players for both groups between U11 and U15. These results suggest important information regarding the need to design interventions to reduce body fat mass particularly in overweight and obese rugby players. Conflicting results have been reported in the literature regarding the evolution of BFMI during adolescence in the general population [22, 25–29]. However, in the only available longitudinal study, a decrease in BFMI has been observed during adolescence in American boys, reaching a minimum level at the age of 15 years old [26]. It is important to note that FFMI increased significantly between age categories and by the same extent for both groups (around + 16%). This pattern of variation is characterized by a small increase (NW, + 2.8%; OW/OB, + 4.2%) between U11 and U13 and a larger increase during the transition between U13 and U15 (NW, + 13.5%; OW/OB, + 11.3%). These results are consistent with the previous findings indicating that the increase in BMI during growth is mainly attributable to the increase in fat-free mass in adolescent boys [29]. Moreover, it is well accepted that a marked increase in fat-free mass is observed from the age of peak height velocity (on average at about 14 years in boys). Nevertheless, BMI and BFMI did not change significantly for both groups during the transition from U11 to U13. The comparison of the present results with previous studies is difficult because, to our knowledge, no study has evaluated BFMI and FFMI in young athletes during growth. In our study as a whole, average FFMI/BFMI and %BF values were 16.3/5.2 kg m^−2^ and 23.3%, respectively. Lower values in non-athletes children and adolescents for similar age categories have been reported in America [26], Iran [28], and China [30]. However, in 12-year-old Brazilian adolescent boys [31], BFMI (4.8 kg m^−2^) and the prevalence of obesity (10.3%) were similar to those of our U13 players (4.7 kg m^−2^; 9.7%), but with lower FFMI. Korean boys [32] had higher BFMI (5.3 kg m^−2^) and %BF (27%) with a similar FFMI (14.3 kg m^−2^) compared to the values observed for our U11 players (FFMI/BFMI 14.9/4.4 kg m^−2^; %BF 21.5%). In the present study, the correlations between BMI and %BF ranged from 0.79 to 0.87 across age categories, improving to 0.91–0.96 when using BFMI. These high correlations between BMI, %BF, and BFMI have been reported in adolescents who had higher BMI measures [26, 27, 30].

Body mass and body size are important components of performance in rugby [14]. In our study, the average BMI for U13 is very close to the data reported in Australia adolescent rugby players [14] in a similar category (20.7 kg m^−2^). In contrast, the average BMI was higher for our U15 (25.1 versus 21.7 kg m^−2^), solely due to a greater body mass (72.8 versus 64.3 kg). In our sample, the young rugby players did not undertake any specific strength training in their clubs. Thus, it is unlikely that this difference was attributable to fat-free mass differences but rather to fat mass differences. Specialization according to playing position (forwards-backs) is not yet defined in France in these age categories. Although overweight/obese players had higher BFMI (range, + 2.9–3.7 kg m^−2^) than normal-weight in the present study, they also had higher FFMI (range, + 2.4–2.7 kg m^−2^). It is important to mention that the majority of obese rugby players (from 66 to 100%) were in the highest tertile for BFMI and FFMI. Moreover, we found moderate correlations for U11 and U13 and strong correlation for U15 between BFMI and FFMI (Fig. 2), indicating a dependence between these two components of body composition. It was suggested that being overweight could contribute to a greater fat-free mass to support an excess body weight [22]. This can be particularly true for adolescents practicing a sport such as rugby by stimulating body weight during force and power exercises. This relationship between BFMI and FFMI was weak or moderate in the general population of adolescents [27, 28]. Interestingly, Infante et al. [33] did not find any correlation between BFMI and FFMI in male rugby players competing in the regional league, with a high prevalence of overweight and obesity (79%). This conflicting observation could be partly related to a small sample size, age differences, and maintained excess body fat over a period of time. Thus, as illustrated in Table 3, it seems difficult to dissociate the elevated fat mass from the elevated fat-free mass and inversely, except for the U11 overweight/obese players in which the relationship between BMI and FFMI was weak. The latter result has been observed in the general population among relatively heavy adolescents (> 85th percentile) [27]. This suggests that the difference in BMI between young rugby players was not only due to fat mass but also to a difference in fat-free mass. On the other hand, BMI can be a useful tool to evaluate adiposity for high levels of BMI in the U11 rugby players. Ode et al. [34] pointed out that BMI incorrectly classified athletes with normal body fat mass as overweight due to a large muscle mass. It is important to indicate that a given BMI may correspond to different combinations of FFMI and BFMI in our study. For example, among U15, two players with similar BMI (22.4 and 22.3 kg m^−2^) had a BFMI of 4 and 6.6 kg m^−2^, with a FFMI of 18 and 15.7 kg m^−2^. So, chart analysis of BFMI and FFMI according to the 25–75th percentile distribution may be helpful to determine the contribution of body fat mass and fat-free mass to BMI and to avoid misclassification. Our results pointed out that BMI overestimated body fat mass for one player classified as overweight but having high fat-free mass with low-fat mass by BFMI. Inversely, an underestimation of body fat mass by BMI has been observed for two players classified as normal-weight by BMI (U11 and U13), but having low fat-free mass with high-fat mass by BFMI. Interestingly, none of U15 was in this situation. Using this method, Nakao and Komiya [19] considered children as obese in this condition. An interesting question is how to reach an optimal body composition for high BMI levels for players who will be likely to play as forwards. In the present sample, the BFMI of overweight and obese players was from 83.3 to 100% higher compared to normal-weight players, while FFMI was higher by only from 16.4 to 18.5%. It means that a higher BMI in obese and overweight players was mainly due to a higher body fat mass compared to normal weight players. In the same way, Darral-Jones et al. [35] pointed out that the adiposity of forwards was 71% higher compared to backs in adolescent rugby union players. Therefore, young rugby players classified as overweight and obese in our study predestine them to become forwards. Moreover, this aspect should be considered because lower adiposity levels throughout junior age categories may partially contribute to long-term career progression [36]. Although fat mass can take on a positive role during physical collisions as a protective effect, it has been suggested that a higher fat mass could be associated with greater injury rates [37] and had a significant impact of the locomotor profile of the players [35]. Our results highlight the issue of children promoted to a higher age band in sports such as rugby, where major differences in body weight and body composition already exist between players in the same age category. For instance, in the present study, the highest and the lowest body mass for U11 were 87.2 and 22.7 kg, respectively, with a large coefficient of variation for this parameter (24%). This extreme situation in terms of body mass between young rugby players has lead Australia’s federation to modify their original policies, by authorizing smaller players to be exempted and compete with younger players in order to ensure their safety and to increase participation to this sport [37]. This aspect should be considered by the French Rugby Federation in which young rugby players are graded by age. In relation to this discussion, the desire to increase body mass among adolescent rugby players in order to increase power and strength can cause confusion between the concepts of body mass and body composition. Moreover, an increase of muscle mass requires an adequate resistance training program combined with a diet that supplies high energy and specific protein to produce the desired gains. In this context, young athletes are often vulnerable to misinformation about muscle growth and development because of a lack of accurate information regarding dietary requirements for muscle gain [2]. Furthermore, resistance training program with weight lifting or body weight exercises is recommended at the end of the puberty. Thus, the major risk in young rugby players is that excess calories are ingested compared to the energy spent, leading to the storage of fat in the adipose tissue. In this context, Thivel et al. [38] in France and Walsh et al. [39] in Ireland have pointed out the necessity to improve nutritional education in adolescent rugby players. Educating younger players and parents and raising awareness about the positives and negatives of increased size from fat-free mass versus fat mass are also of fundamental importance.

Methodological considerations should be mentioned to interpret our findings. Firstly, maturation level has not been determined in our population. Malina et al. [4] observed that more than half young American football players classified in advanced maturity were obese. As mentioned in the introduction, the age categories of the French Rugby Federation have been used in our analysis in order to reflect on the differences in body composition within the constraints of real competition. Secondly, although skinfold measurements have been conducted by the same investigator to assess the body composition in this large sample of young rugby players, several sources of measurement error are mentioned in the literature, particularly in fatter adolescents. This two-component model assuming that fat and fat-free mass have a fixed density does not take into account age differences and individual variability for this parameter [20]. In addition, the determination of bone mineral density is not possible by this method as opposed to the whole body densitometry. Hence, a higher bone content and density in obese children and adolescents compared to normal-weight peers should also be taken into account [40]. Finally, we have conducted a cross-sectional study in which data is collected at a single point in time for each age category in different players. A longitudinal study is more powerful because the changes of body composition across the time span of interest are within the same players.

Findings from this study point out some practical applications. Our results highlight the importance of specific interventions before 13 years to prevent the increase of body fatness across age categories, especially in rugby players who are considered as overweight or obese. The increase in fat mass during pubescence could have a negative effect on performance and on health status in the future. Calculating BMI for young rugby players in clubs to detect overweight and obesity must be used with caution due to significantly moderate and strong correlations between BMI, BFMI, and FFMI, and to different combinations of FFMI and BFMI that result in the same BMI. Thus, these indexes could be used to distinguish the contribution of body fat mass and fat-free mass to changes in body composition across age categories in young rugby players classified as overweight, obese, and normal-weight by BMI. Moreover, the chart analysis of these indexes is relevant to identify individually the morphotype of the player. Coaches should clearly define objectives in body composition control and body fatness reduction in young rugby players.

## Conclusions

In conclusion, 32.8% of young rugby players aged 9–14 years were considered as overweight and 12.8% were classified as obese according to the IOTF criteria. However, BMI can overestimate an excess of adiposity for a player having a high fat-free mass classified as obese or overweight. Thus, the drastic increase in the prevalence of overweight and obesity during the transition from U13 to U15 needs to be put into perspective because the increase in BMI was explained by the increase of both fat mass and fat-free mass in overweight and obese rugby players. Indeed, 53% of young players classified as obese and overweight by BMI had an excess body fat by using BFMI above the 75th percentile. In normal-weight players, the increase in BMI from U11 to U15 was mainly attributable to the changes of fat-free mass. An interesting follow-up to this study would be to look at a professional rugby club with an academy and see if they have data on the progression of athletes through their academy to their first team to look at the relationship of BMI with progression to high performance.

## Abbreviations

%BF: Percentage of body fat
BFMI: Body fat mass index
BMI: Body mass index
FFMI: Fat-free mass index
NW: Normal-weight
OW/OB: Overweight/obese
U11: Under 11 years
U13: Under 13 years
U15: Under 15 years
